# Supplementary material for: Emergent and robust ferromagnetic-insulating state in highly strained ferroelastic LaCoO3 thin films
Source: Nat Commun. 2023 Jun 19;14:3638. doi: 10.1038/s41467-023-39369-6 (PMC10279738; doi:10.1038/s41467-023-39369-6)
Supplement: Supplementary file 1 — Supplementary Information [file 41467_2023_39369_MOESM1_ESM.pdf]

Supplementary Information

Emergent and Robust Ferromagnetic-Insulating State in Highly Strained Ferroelastic $LaCoO_3$ Thin Films

Dong Li[1†], Hongguang Wang[2†*], Kaifeng Li[1†], Bonan Zhu[3*], Kai Jiang[4,5*], Dirk Backes[6], Larissa S. I. Veiga[6], Jueli Shi[7], Pinku Roy[8,9], Ming Xiao[10], Aiping Chen[8], Quanxi Jia[9], Tien-Lin Lee[6], Sarnjeet S. Dhesi[6], David O. Scanlon[3,6], Judith L. MacManus-Driscoll[10], Peter A. van Aken[2], Kelvin H. L. Zhang[7*], and Weiwei Li[1*]

1. College of Physics, MIIT Key Laboratory of Aerospace Information Materials and Physics, State Key Laboratory of Mechanics and Control for Aerospace Structures, Nanjing University of Aeronautics and Astronautics, Nanjing 211106, China
2. Max Planck Institute for Solid State Research, Heisenbergstr. 1, 70569, Stuttgart, Germany
3. Department of Chemistry, University College London, London WC1H 0AJ, UK
4. Department of Materials, East China Normal University, Shanghai 200241, China
5. School of Arts and Sciences, Shanghai Dianji University, Shanghai 200240, China
6. Diamond Light Source Ltd., Harwell Science and Innovation Campus, Didcot, Oxfordshire OX11 0DE, UK
7. State Key Laboratory of Physical Chemistry of Solid Surfaces, Collaborative Innovation Center of Chemistry for Energy Materials, College of Chemistry and Chemical Engineering, Xiamen University, Xiamen 361005, China
8. Center for Integrated Nanotechnologies (CINT), Los Alamos National Laboratory, Los Alamos, New Mexico 87545, USA
9. Department of Materials Design and Innovation, University at Buffalo-The State University of New York, Buffalo, NY 14260, USA
10. Department of Materials Science and Metallurgy, University of Cambridge, Cambridge CB3 0FS, UK

Corresponding authors: hgwang@fkf.mpg.de, bonan.zhu@ucl.ac.uk, kjiang@ee.ecnu.edu.cn, kelvinzhang@xmu.edu.cn, wl337@nuaa.edu.cn

[†]These authors contribute equally to this work

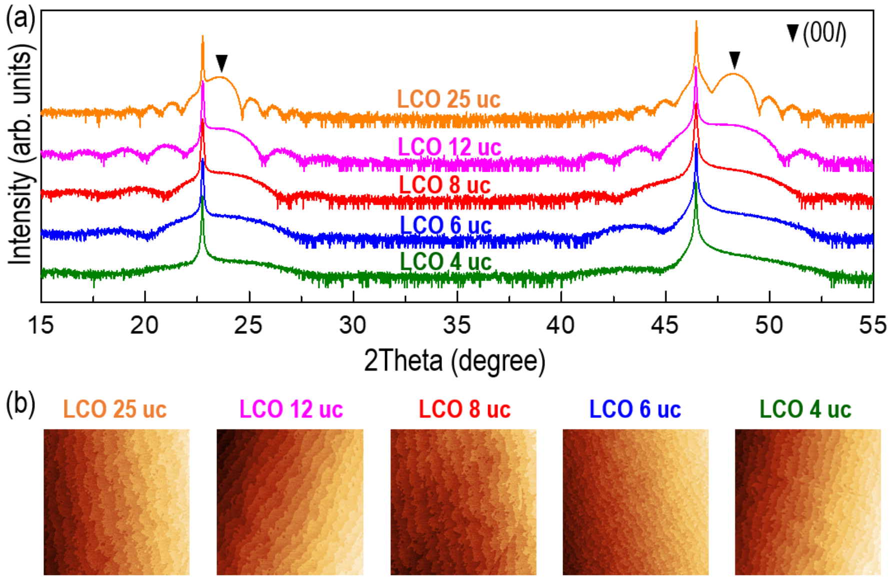

Supplementary Figure 1 Structural analysis by XRD and AFM. (a) XRD θ-2θ scans of LCO thin films with different thickness. "▼" was used to label the (00l) peaks of LCO thin films. (b) Typical 5 × 5-μm AFM height images of LCO thin films with different thickness.

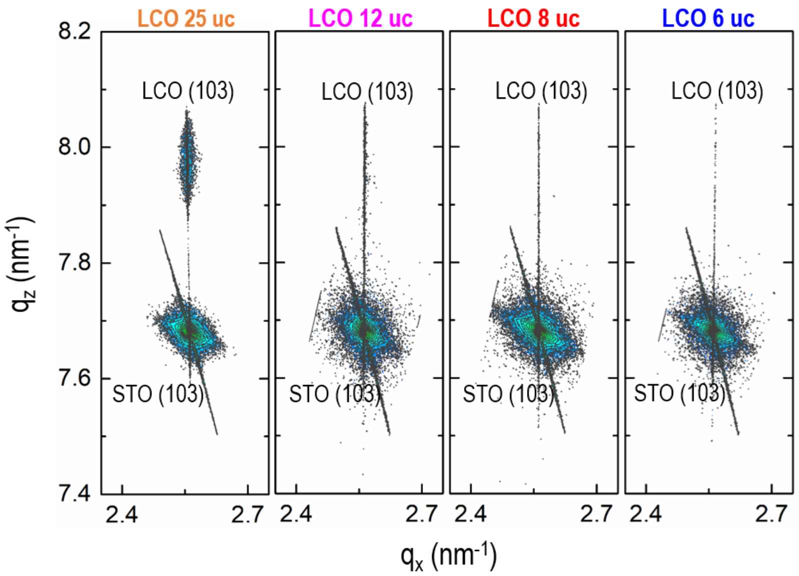

Supplementary Figure 2 Strain analysis by reciprocal space maps. Reciprocal space maps of (103) Bragg reflections of STO and LCO for LCO thin films with different thickness.

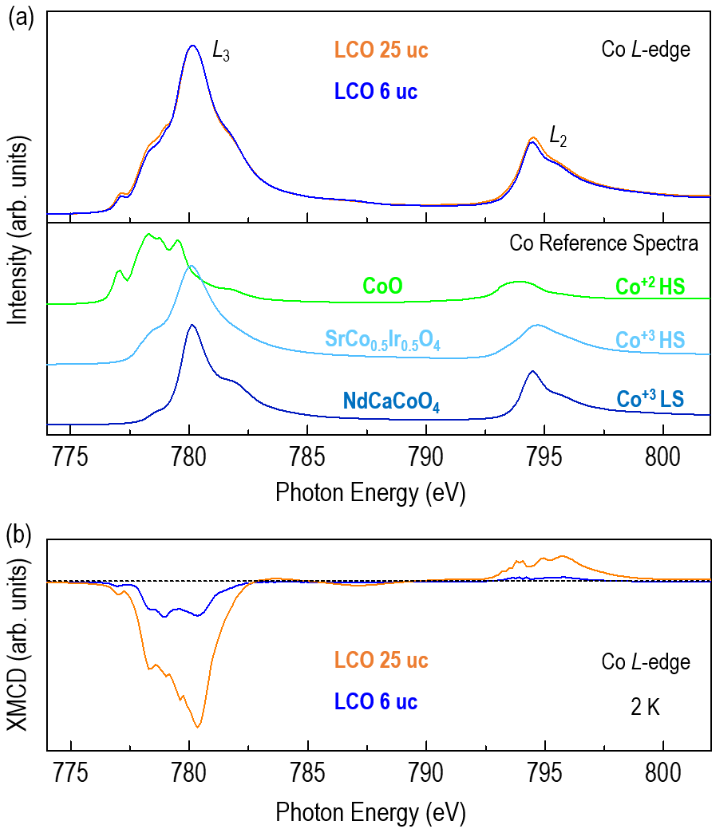

Supplementary Figure 3 Spin states and magnetic properties of LCO films. (a) XAS spectra (top panel) for LCO 25 uc and 6 uc, and reference spectra (bottom panel) for Co^{2+} HS, Co^{3+} HS, and Co^{3+} LS. (b) XMCD spectra for LCO 25 uc and 6 uc.

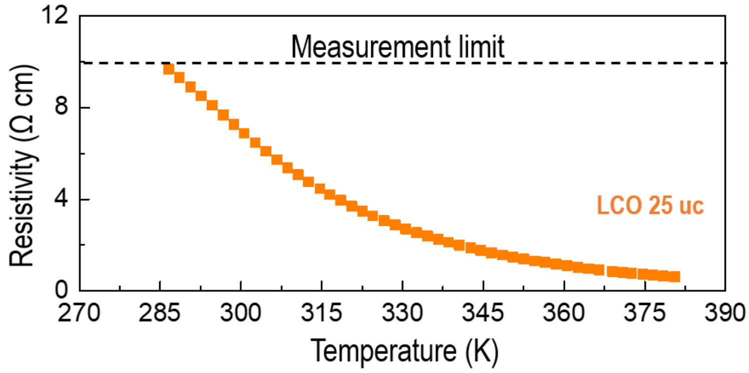

Supplementary Figure 4 Transport property of LCO film. Temperature-dependent resistivity of LCO 25 uc.

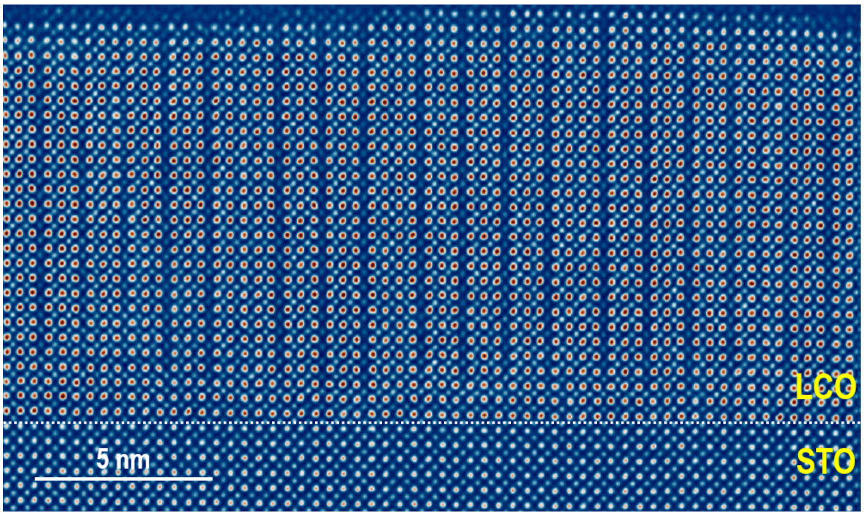

Supplementary Figure 5 Structural analysis by STEM. Z-contrast HAADF-STEM image of LCO 25 uc in cross-sectional orientation, projected along the [010] zone axis.

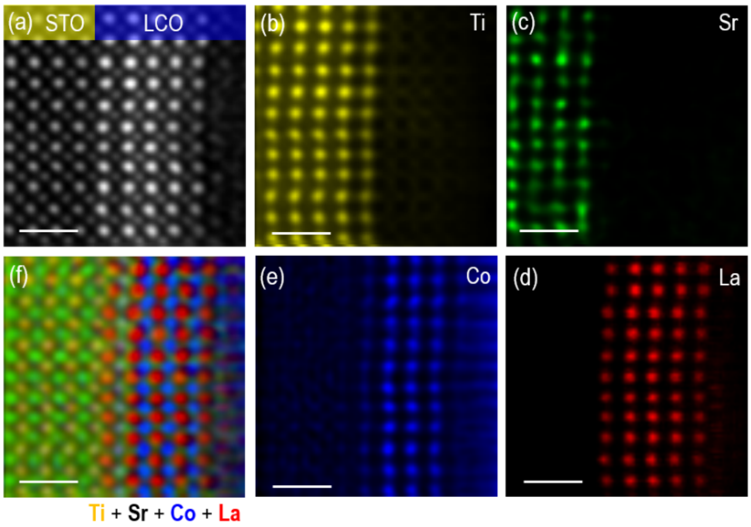

Supplementary Figure 6 Structural analysis by STEM and EELS. (a) Z-contrast HAADF-STEM image of LCO 6 uc in cross-sectional orientation, projected along the [010] zone axis. (b-f) Atomically resolved STEM-EELS elemental maps of Sr, Ti, La, and Co. Scale bar: 1 nm.

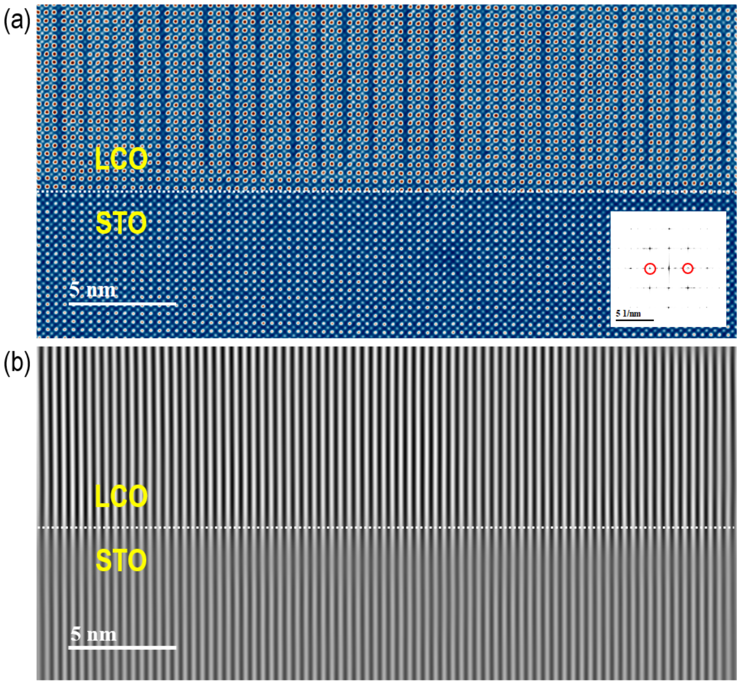

Supplementary Figure 7 STEM investigation of interfacial lattice structure. (a) The atomic resolution HAADF-STEM image of the interface between LCO and STO, the inset is the FFT image. (b) the corresponding inverse FFT image using the reflections marked by red circles in the inset of (a).

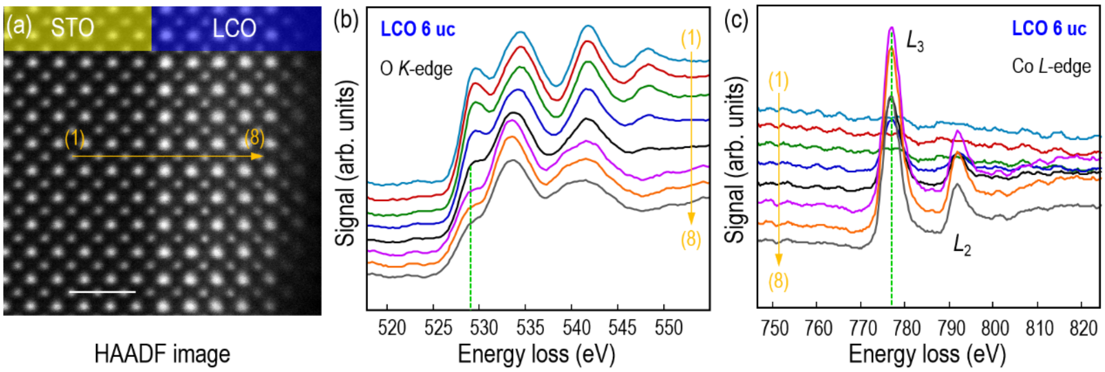

Supplementary Figure 8 EELS fine structures of LCO film. (a) Z-contrast HAADF-STEM image of LCO 6 uc in cross-sectional orientation, projected along the [010] zone axis. Scale bar: 1 nm. (b) O *K*-edge and (c) Co *L*-edge spectra of LCO 6 uc measured along the yellow arrow direction in (a).

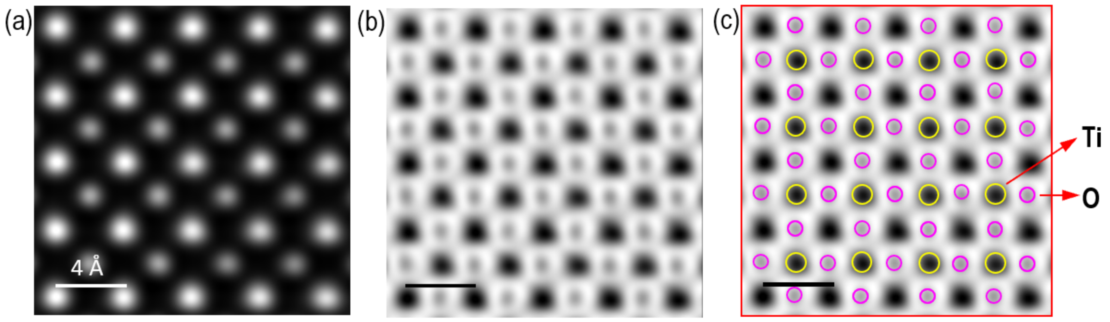

Supplementary Figure 9 STEM investigation of oxygen octahedra in STO. Simultaneously acquired HAADF (a) and ABF (b) images of the standard STO. (c) The corresponding ABF image (b) with fitted oxygen (red circles) and Ti positions (yellow circles).

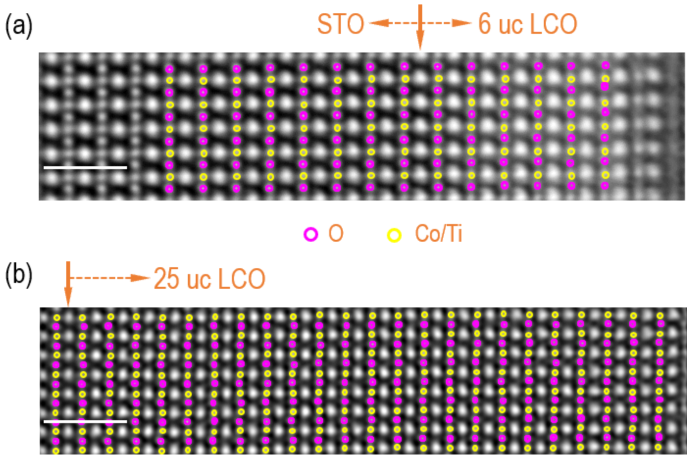

Supplementary Figure 10 STEM investigation of oxygen octahedra in LCO. Inverted ABF image of 6 uc LCO (a) and 25 uc LCO (b) with fitted position of oxygen ions (red circles) and Co (Ti) ions (yellow circles).

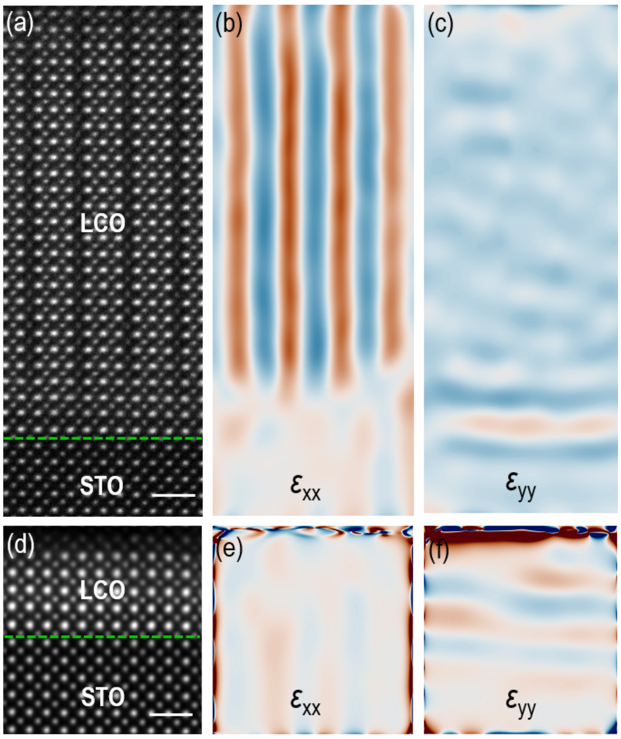

Supplementary Figure 11 Strain analysis by STEM. Z-contrast HAADF-STEM images of (a) LCO 25 uc and (d) LCO 6 uc in cross-sectional orientation, projected along the [010] zone axis. Scale bar: 1 nm. Respective in-plane (ε_{xx}) (b) and out-of-plane (ε_{yy}) (c) strain maps obtained from STEM-based GPA for LCO 25 uc. Lattice expansion is clearly observed along the dark stripes in (b). Respective in-plane (ε_{xx}) (e) and out-of-plane (ε_{yy}) (f) strain maps obtained from STEM-based GPA for LCO 6 uc. The lattice of the STO substrate was taken as a reference for strain analysis.

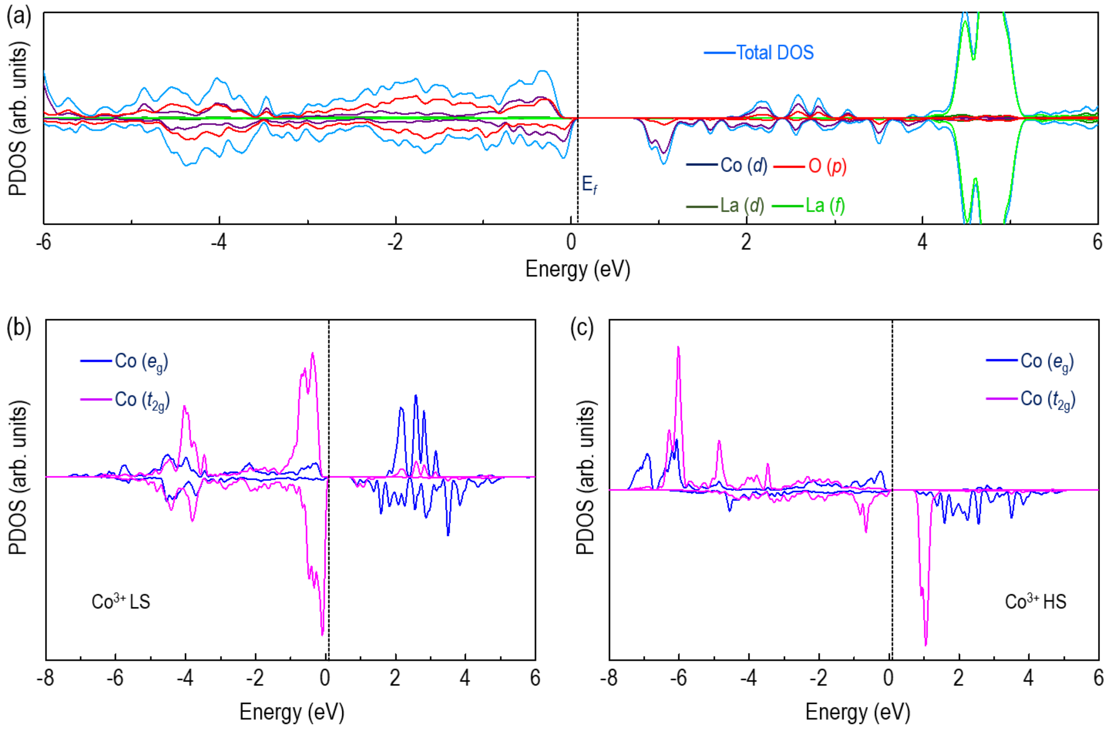

Supplementary Figure 12 Projected density of states in LCO from DFT calculations. Projected density of state (PDOS) spectrum for (a) LCO without ordered V_O or dark stripes, (b) Co ions with low-spin (LS) states, and (c) Co ions with high-spin (HS) states.

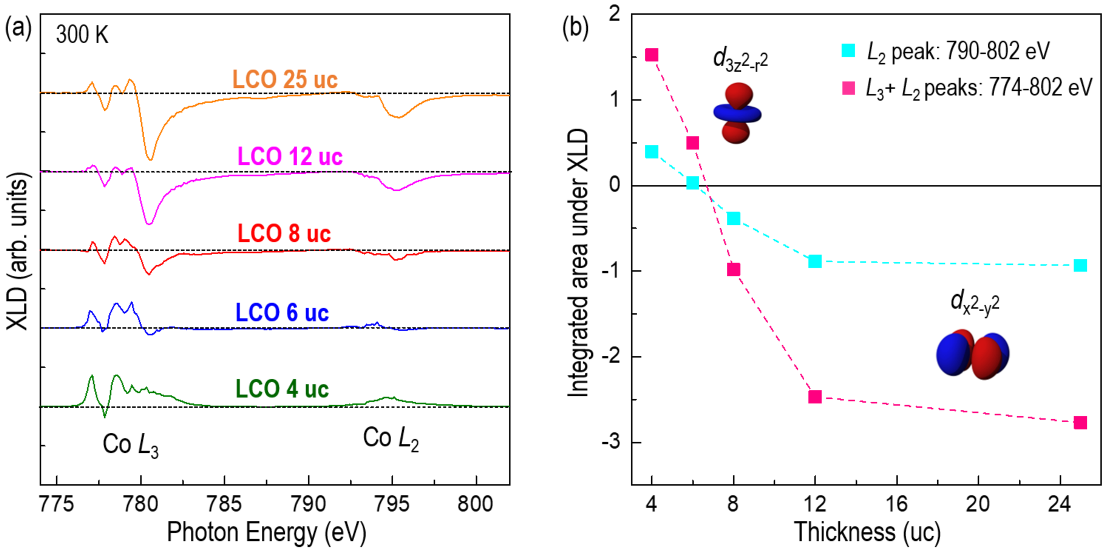

Supplementary Figure 13 Orbital occupancy of LCO. (a) XLD spectra of LCO thin films with different thickness. (b) Integrated area of XLD signal as a function of thickness. The unexpected out-of-plane $d_{3z^2-r^2}$ occupancy in e_g orbital (observed in LCO thin film with a thickness below 6 uc) under tensile strain can be explained by a vacuum-interface induced extra contribution of the orbital configuration, favoring occupancy of the out-of-plane $d_{3z^2-r^2}$ states[1].

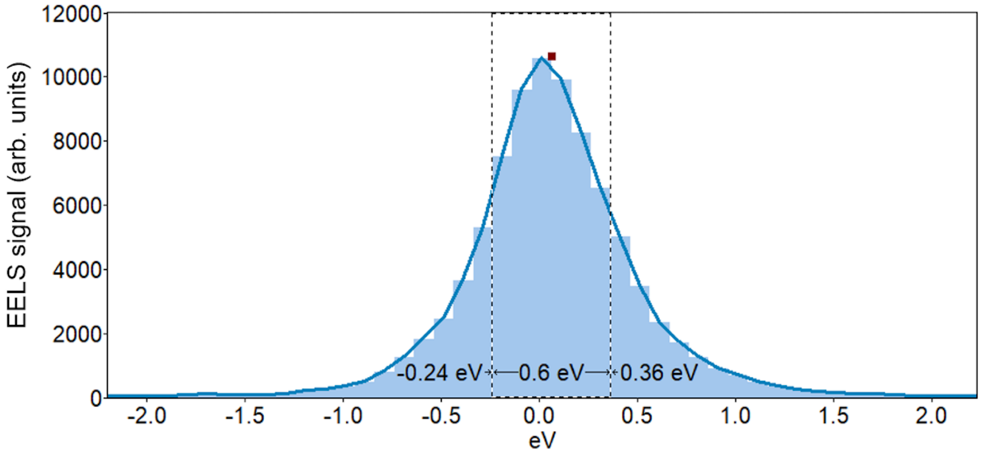

Supplementary Figure 14 Energy resolution of EELS. Simultaneously acquired zero-loss spectrum during EELS line scan (Figure 2). The estimated energy resolution is about 0.6 eV.

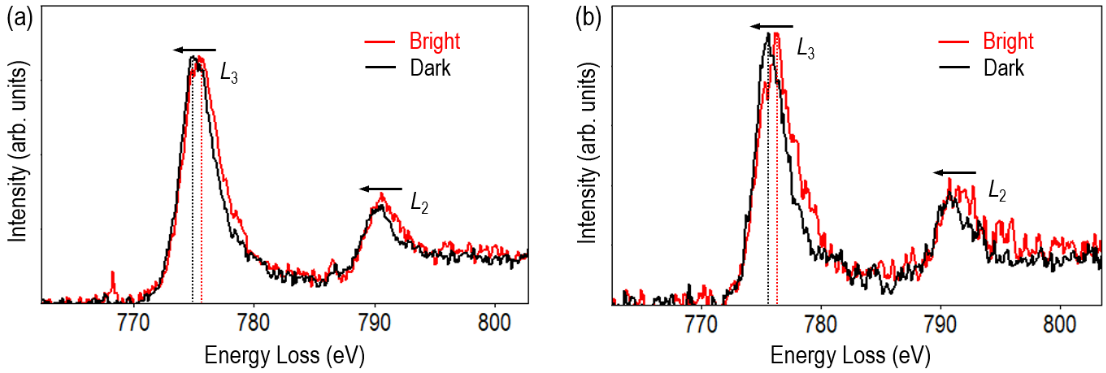

Supplementary Figure S15 EELS investigation with different electron dose conditions. Co-$L_{2,3}$ spectra of dark stripes and bright stripes measured with (a) high- and (b) low-dose conditions in 25 uc LCO. For the high-dose condition, the EELS spectrum was obtained by exposing for a long time at a specific position (25 sec/1 point). For comparison, a low-dose EELS spectrum was achieved by integrating short-exposure-time spectra obtained at equivalent but various points (0.1 sec/1 point × 20 points).

Reference

[1] Pesquera, D. et al. Surface symmetry-breaking and strain effects on orbital occupancy in transition metal perovskite epitaxial films. *Nat. Commun.* **3**, 1189 (2012).
